# Supplementary material for: Causal Relationship between Aging and Anorexia Nervosa: A White-Matter-Microstructure-Mediated Mendelian Randomization Analysis
Source: Biomedicines. 2024 Aug 16;12(8):1874. doi: 10.3390/biomedicines12081874 (PMC11351342; doi:10.3390/biomedicines12081874)
Supplement: Supplementary file 1 [file biomedicines-12-01874-s001.zip › Supplementary Figure/Figure S8/Leaveoneout plot of MR analysis for TL to ubm-b-1793.pdf]

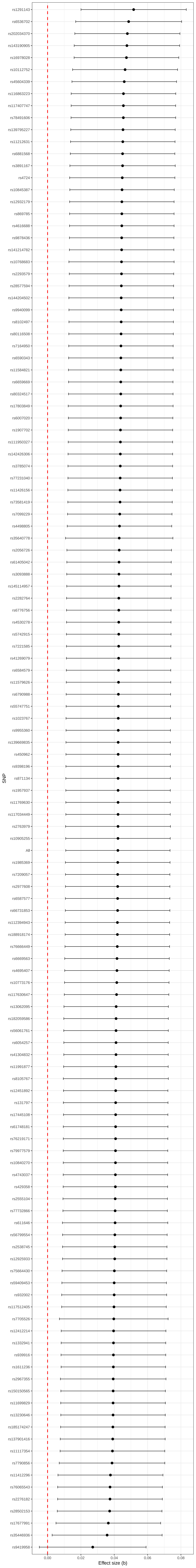
rs1291143
rs6536702
rs202034370
rs143190905
rs16978028
rs10112752
rs45604339
rs116863223
rs117407747
rs78491606
rs139795227
rs11212631
rs6881568
rs3891167
rs4724
rs10845387
rs12932179
rs869785
rs4616688
rs9878436
rs141214782
rs10768683
rs2293579
rs28577594
rs144204502
rs9940099
rs8102497
rs80116508
rs7164950
rs6590343
rs11584821
rs6659669
rs80324517
rs17803849
rs6007020
rs1907702
rs111950327
rs142426306
rs3785074
rs77231040
rs11426156
rs73581419
rs7099229
rs4498805
rs35640778
rs2056726
rs61405042
rs3093888
rs145114957
rs2282764
rs6776756
rs4530278
rs5742915
rs7221585
rs41269079
rs6584579
rs11579626
rs6790988
rs55747751
rs1023767
rs9955360
rs139669835
rs450962
rs9398196
rs871134
rs1957937
rs11769630
rs117034449
rs2763979
rs10905255
All
rs1985369
rs7209057
rs2977608
rs6587577
rs66731853
rs112394943
rs188918174
rs76666449
rs6669563
rs4695407
rs10773176
rs117630647
rs13062095
rs182059586
rs56061761
rs6054257
rs41304832
rs11991877
rs8105767
rs12451892
rs131797
rs17445108
rs61748181
rs76219171
rs79977579
rs10840270
rs4743037
rs429358
rs2555104
rs77732866
rs611646
rs56799554
rs2538745
rs12925933
rs75664430
rs59409453
rs932002
rs117512405
rs7705526
rs12412214
rs1332941
rs939916
rs1611236
rs2967355
rs150150565
rs11699829
rs13230646
rs185174247
rs137901416
rs11117354
rs7790856
rs11412296
rs76065543
rs2276182
rs28502153
rs17677991
rs35446936
rs9419958
SNP
0.00
0.02
0.04
0.06
0.08
Effect size (b)
